# The impact of body mass index on mortality in patients with acute kidney injury: a systematic review protocol

**DOI:** 10.1186/s13643-018-0825-3

**Published:** 2018-10-22

**Authors:** Maria P Barrett, David Moore, Fang Gao Smith, Indranil Dasgupta

**Affiliations:** 1 Dietetics Department, Therapy Services Unit, Manchester University NHS Foundation Trust, Oxford Road, Manchester, M13 9WL UK; 2 Institute of Applied Health Research, University of Birmingham, Birmingham, B15 2TT UK; 3 Institute of Inflammation and Ageing, University of Birmingham, Birmingham, B15 2TT UK; 4 Heartlands Hospital, University Hospitals Birmingham NHS Foundation Trust, Bordesley Green East, Birmingham, B9 5SS UK

**Keywords:** Acute kidney injury, AKI, Body mass index, BMI, Mortality, Systematic review, Protocol

## Abstract

**Background:**

Acute kidney injury (AKI) refers to a broad spectrum of kidney damage and is attributed a high morbidity and mortality rate at all degrees of severity. Obesity increases the risk for developing AKI. However, some studies have shown that obesity at onset of AKI is paradoxically associated with greater survival. The aim of this review is to explore the relationship between body mass index and survival in patients with AKI.

**Methods:**

An electronic search will be conducted using MEDLINE, EMBASE, CINAHL and CENTRAL using predefined search strategies. The cited and citing references of selected key studies will also be searched for relevant articles. Risk of bias will be assessed using a modified Quality in Prognosis Studies (QUIPS) tool. The primary outcome will be an exploration of the association between BMI and mortality in patients presenting with AKI. Two authors will independently select, data extract, and risk of bias assess articles. Any discrepancies will be resolved by consensus or by consulting a third author. A narrative synthesis of the findings from the included studies will be presented. Meta-analyses will be conducted where the data is available from clinically and methodologically similar studies and in the same format. Heterogeneity in such analyses, beyond that expected by chance, will be quantified using the *I*^2^ statistic. Sub-group analyses will be performed to determine the influence of gender, AKI duration, underlying aetiology, and intervening treatments, on pooled results.

**Discussion:**

Body mass index may be an important modifiable risk factor for mortality in patients presenting with AKI. The proposed systematic review will help to elucidate the association between all categories of BMI and survival in this patient group.

**Systematic review registration:**

PROSPERO CRD42017071124.

**Electronic supplementary material:**

The online version of this article (10.1186/s13643-018-0825-3) contains supplementary material, which is available to authorized users.

## Background

Acute kidney injury (AKI) describes a rapid onset of kidney function impairment, denoted by a significant drop in renal function seen typically over a 48-h period. It is characterised by an acute rise in serum creatinine and reduction or absence of urine output. The term AKI has replaced acute renal failure, for which there is no standardised definition. It can be used to refer to a broad spectrum of kidney damage and is attributed a high morbidity and mortality rate at all degrees of severity [1].

Conflicting evidence exists regarding the potential impact of body mass index (BMI) on outcomes in patients with AKI. Chao et al. (2014) showed a U-shaped association between BMI and mortality in geriatric critically ill AKI patients (for obese versus normal: [HR, 1.22, CI 1.01–1.49, *p* = 0.042]; for underweight versus normal [HR, 1.60, CI 1.05–2.61, *p* = 0.038]), Danzinger et al. (2016) showed that within-hospital mortality increased with each 5 Kg/m^2^ increment in BMI (adjusted OR, 1.10, CI 1.06–1.24, *p* < 0.001), while Kim et al. (2017) showed an inverse relationship with mortality (adjusted HR, 0.94 [CI 0.90–0.98], *p* = 0.01) between patients in the highest BMI tertile (25.5–37.1 Kg/m^2^) compared to those in the lowest tertile (13.5–21.8 Kg/m^2^) [2–4]. The difference in these results might be explained by different ethnic populations studied, highlighting the potential impact of ethnicity and body composition on outcomes. The paradoxical relationship shown in some studies between obesity and survival has also been noted in patients with CKD [5]. This is thought to be secondary to several factors including a different metabolic profile compared to healthy weight patients, greater lean body mass, and sequestration of uraemic toxins in adipose tissue [6, 7].

The aim of this review is to explore the relationship between BMI and survival in patients with AKI. To the authors’ knowledge, there is currently no other systematic review published or planned which attempts to summarise the evidence for whether body mass has a role in determining mortality in this patient group. The precipitating factors, aetiology and degrees of severity for AKI are multifarious [8]. This, arguably, makes grouping of studies difficult but as small decrements in kidney function are known to be of substantial clinical significance, the incidence of all-cause mortality and its association with body mass, despite the stage of AKI, will be the primary outcome of this review. It is hoped that in summarising the current evidence, this systematic review will inform practice and future trial design.

### Objectives

The present systematic review will aim to assess whether a similar obesity paradox exists in adult patients with AKI as has been seen in patients with CKD. More specifically, the review aims to answer the following research question: In adult patients with AKI, is there an association between BMI, at onset of AKI, and mortality?

## Methods/design

### Exposure

Adult studies that include BMI measurements for all in-patients at presentation of AKI and mortality incidence/risk will meet the exposure criteria.

### Study design

Any design analysing longitudinal data. This will include prognostic studies that, for example, have predicted risk of mortality in patients with AKI based on presenting characteristics, including BMI. Observational and case-control studies which have recorded time-to-death data, BMI and AKI occurrence (and ideally AKI stage) will also be included.

### Population

Adult in-patients (aged 18 years and above) with AKI. Any study which includes a mix of ages (i.e. participants are aged both < 18 years and above) but allows the extraction of data specifically for participants > 18 years will be included.

### Outcome

Data, of any format, are provided on the association between BMI (measured upon presentation of AKI and analysed as either a continuous variable or a categorical variable) and mortality (incidence or risk).

### Search methods for identification of studies

We will search the following databases for relevant studies with no language or date constraints: MEDLINE, EMBASE, CINAHL and Cochrane Central Register of Controlled Trials (CENTRAL).

The search strategy for all databases will combine free text and where available index terms (e.g. MeSH). Key search terms will include the following: (“acute kidney injury” or “acute renal failure”) and (“body mass index”, “BMI”, “obes*”, “*weight”) and (“mortality” or “survival”).

The aim of our search strategy is to yield adult studies (age 18 and above) as well as studies of mixed ages that allow the extraction of data for adult patients only. Table 1 includes the index list for each database search strategy and the time period covered by that database. It is expected that a broad list of studies will be returned so that all studies which include the data of interest for this review can be found. The authors acknowledge that MEDLINE defines the term “adult” as being aged 19 years or over. For this reason, the search terms “18 year*” and “age* 18” will be searched in all fields (af) and combined with “exp Adult/ or adult.mp”, where “exp” denotes a search term that automatically includes closely related indexing terms and “mp” describes a term that appears in the title, abstract and subject heading. The search strategies used by Kastner et al. (2006) when reviewing the sensitivity of MEDLINE for extracting age-specific studies were reviewed and informed the strategy used in this MEDLINE search [9]. Similarly, the Cinahl database search has been expanded to combine the terms “((18 or eighteen) ADJ3 (age* OR year)).af” with “adult.af” and “exp adult/”, to increase the sensitivity of the search. Here, “ADJ3” describes a search technique that finds terms in any order with two words (or fewer) between them.

**Table 1:** Search strategy

Database	Search strategy
EMBASE (1974 to present)	1. Acute kidney injury.mp. or acute kidney failure/
	2. body mass.mp. or body mass/
	3. obesity/ or morbid obesity/
	4. obesity.mp.
	5. morbid obesity.mp.
	6. sarcopenic obesity.mp. or sarcopenic obesity/
	7. overweight.mp.
	8. underweight.mp. or underweight/
	9. body weight.mp. or body weight/
	10. mortality/ or mortality.mp.
	11. mortality rate/ or mortality rate.mp.
	12. death rate.mp.
	13. survival.mp. or survival/
	14. survival rate.mp. or survival rate/
	15. 2 or 3 or 4 or 5 or 6 or 7 or 8 or 9
	16. 10 or 11 or 12 or 13 or 14
	17. 1 and 15 and 16
	18. limit 17 to (adult < 18 to 64 years> or aged < 65+ years>)
MEDLINE	1. Acute kidney injury.mp. or Acute Kidney Injury/
	2. acute renal failure.mp.
	3. body mass index.mp. or Body Mass Index/
	4. Body Weight/ or body mass.mp.
	5. overweight.mp. or Overweight/
	6. underweight.mp. or Thinness/
	7. Obesity/ or obesity.mp.
	8. morbid obesity.mp. or Obesity, Morbid/
	9. obese.mp.
	10. mortality.mp. or Mortality/
	11. mortality rate.mp.
	12. death rate.mp.
	13. Survival/ or survival.mp.
	14. survival rate.mp. or Survival Rate/
	15. 1 or 2
	16. 3 or 4 or 5 or 6 or 7 or 8 or 9
	17. 10 or 11 or 12 or 13 or 14
	18. 15 and 16 and 17
	19. exp. Adult/ or adult.mp.
	20. ((year* or age*) adj2 eighteen).af.
	21. “18 year* .af.”
	22. “age* 18 .af.”
	23. 19 or 20 or 21 or 22
	24. 18 and 23
CINAHL	1. (“acute kidney injur*”).ti,ab
	2. (“acute renal failure”).ti,ab
	3. “KIDNEY FAILURE, ACUTE”/
	*4. (1 OR 2 OR 3)*
	5. (“body mass index”).ti,ab
	6. (BMI).ti,ab
	7. (“body mass”).ti,ab
	8. (obesity).ti,ab
	9. (obese).ti,ab
	10. (overweight).ti,ab
	11. (underweight).ti,ab
	12. (thin*).ti,ab
	13. (weight).ti,ab
	14. “BODY WEIGHT CHANGES”/
	15. “BODY WEIGHT”/
	16. “BODY MASS INDEX”/
	17. “OBESITY, MORBID”/
	18. OBESITY/
	19. THINNESS/
	*20. (5 OR 6 OR 7 OR 8 OR 9 OR 10 OR 11 OR 12 OR 13 OR 14 OR 15 OR 16 OR 17 OR 18 OR 19)*
	21. (mortality).ti,ab
	22. (survival).ti,ab
	23. MORTALITY/
	24. “HOSPITAL MORTALITY”/
	*25. (21 OR 22 OR 23 OR 24)*
	*26. (4 AND 20 AND 25)*
	27. (adult).af
	28. exp ADULT/
	29. ((18 OR eighteen) ADJ3 (age* OR year*)).af
	*30. (27 OR 28 OR 29)*
	*31. (20 AND 25 AND 26 AND 30)*
Cochrane Central Register of Controlled Trials (CENTRAL)	Acute kidney injury: ti,ab,kw
	Acute kidney failure: ti,ab,kw
	“acute renal failure”: ti,ab,kw
	MeSH descriptor: [acute kidney injury] noexp.
	#1 OR #2 OR #3 OR #4
	Body mass index: ti,ab,kw
	Body weight: ti,ab,kw
	MeSH descriptor: [Body mass index] exp.
	MeSH descriptor: [Body weight] noexp.
	#6 OR #7 OR #8 OR #9
	#5 AND #10

The cited and citing references of included studies that meet the exposure criteria will be assessed to identify further relevant articles that were not identified by the aforementioned databases. Grey literature sources will be searched for conference proceedings and relevant study results. These will include “Copac”, “Google Scholar”, “GreyNet International”, “OpenGrey” and “Proquest Dissertations & Theses”.

### Citation management and screening

Search results will be entered into Mendeley Reference Manager package 2017 and duplicates will be removed. Studies will be screened initially according to title and abstract by two authors independently, and those not meeting the criteria will be discarded. Disagreements will be resolved by discussion and referral to a third author if necessary.

After this initial stage, the full text of all remaining studies will be reviewed by two authors independently for inclusion or exclusion in the final study. As before, disagreements will be resolved by discussion and referral to a third author if necessary.

### Data extraction

Data extraction will be performed independently and in duplicate by two review authors. Predefined data extraction spreadsheets will be used to cumulatively extract study details and compile study data for each individual study (Additional file 1). Pilot extraction of data will be performed on three studies.

The information we record from all studies will include journal citation, study design, the type and setting of the study, patient characteristics, number of participants, AKI diagnostic standard, AKI staging, and BMI categorising ranges or whether BMI is reported as continuous or binary values. The type of modelling or statistical approach will be recorded—for example, hazard ratios (HR), odds ratio (OR), regression coefficients, standardised beta regression coefficients, and standardised mean differences—and any adjustment factors used.

For interventional studies, we will record information on the nature of the intervention(s) in each group (including the treatment of the AKI) and mortality outcome.

Prognostic model studies will be analysed separately, looking at model construction, internal validation and external validation. It is anticipated that such studies do not exist.

In the absence of any models, the ideal would be studies within an individual severity of AKI looking at BMI and its association with mortality or a study covering multiple stages of AKI with an association between BMI and mortality described at every stage; stage of disease is accounted for in other factors and BMI is adjusted by stage of disease.

Efforts will be made to contact the authors of primary studies to provide missing data where necessary. If a response is not obtained within 4 weeks, a follow-up email will be sent with a response awaited within 2 weeks. We will not chase beyond that.

### Assessment of risk of bias

It is not expected that any prognostic model studies will be identified. In the event that such a study is found, the Checklist for critical Appraisal and data extraction for systematic Reviews of prediction Modelling Studies (CHARMS) tool will be used to appraise for quality and risk of bias [10].

For all other studies, data will be treated as observational and risk of bias will be assessed through use of the QUIPS (QUality In Prognosis Studies) tool. This is a prognostic risk of bias tool and has been adapted to suit the requirements of this review (Additional file 2) [11]. This tool specifically considers confounding factors like intervention and underlying clinical condition. It has six domains:

1. Study participation
2. Study attrition
3. Prognostic factor measurement
4. Outcome measurement
5. Study confounding
6. Statistical analysis and reporting.

For each domain, specific guidance is given on how to rate the adequacy of reporting by a study as yes, partial or no. We will assign a judgement regarding the risk of bias as high, moderate or low. The QUIPS spreadsheet will be used for entry of risk of bias assessments. Two review authors will independently complete the QUIPS assessment for each study.

We will attempt to contact the trial corresponding author for clarification when insufficient detail is reported to assess risk of bias. Once we have consensus on the quality assessment of the six domains for eligible studies, we will assign them to the following categories:

Low risk of bias: describes studies for which all domains are scored as “yes”.

Moderate risk of bias: describes studies for which one or more domains are scored as partly or one domain is scored as “no”.

High risk of bias: describes studies for which more than one domain is scored as “no”.

The rating of the overall quality of the evidence from this review will be undertaken in consideration of current guidance on the use of the GRADE (Grading of Recommendations, Assessment, Development and Evaluations) approach applied to prognostic studies [12].

The quality and bias risk of included studies will be independently assessed by two authors and verified by a third if necessary.

### Strategy for data synthesis

We will provide a narrative synthesis of the findings from the included studies.

Study authors will be contacted to ideally obtain the raw data of studies reporting on BMI and mortality for patients with AKI. It is hoped this may allow the authors to reclassify the data according to BMI categories (specific to ethnic origin) and mortality risk within that category for each AKI stage.

BMI will be categorised according to the World Health Organisation criteria (underweight: BMI ≤ 19.9 Kg/m^2^; normal weight: 20.0–24.9 Kg/m^2^; overweight: 25.0–29.9 kg/m^2^ of BMI; obese I: 30.0–34.9 kg/m^2^ of BMI; obese II: 35–39.9 Kg/m^2^ of BMI; obese III: BMI ≥ 40 Kg/m^2^) or Asian criteria (underweight: BMI ≤ 18.5 Kg/m^2^; normal weight: 18.5–22.9 Kg/m^2^; overweight: 23.0–24.9 kg/m^2^ of BMI; obese I: 25.0–29.9 Kg/m^2^ of BMI; obese II: BMI ≥ 30 Kg/m^2^), dependent upon the ethnicity of the study population [13, 14]. We will use the generalised least-squares method for trend estimation to calculate the risk of mortality associated with every 5 kg/m^2^ increase in BMI [15, 16].

Where raw data is not available, similar studies will be grouped together. For example, studies will be grouped by in-patient populations (e.g. critically ill vs non-critically ill), AKI staging/ definition (for example, as defined by the Kidney Disease: Improving Global Outcomes [KDIGO] criteria), and by the types of data available (such as hazard ratios [HR], relative risks [RR] and odds ratio [OR]). Categorical and continuous BMI data will be analysed separately (as described above). Studies which report BMI data as binary thresholds or categories will be grouped and used to inform the narrative synthesis.

In order to maximise data for any further analyses, we will consider whether data reported for each study will allow for the calculation of alternate measures, e.g. unadjusted RR and/or ORs from raw data for approximations of unadjusted HRs and associated uncertainty [17, 18]. Any standardised beta regression coefficients from regression models will be converted to odds ratios. Follow-up time over which mortality is reported will be a consideration, particularly when grouping time-to-event data.

The potential for meta-analysis within groups for each type of data will be assessed by considering the methodological (including risk of bias) and clinical similarity of studies. Where meta-analysis is deemed appropriate, the random effects model will be employed due to the likely between study variations. Unadjusted and adjusted data for the same outcome and metric (e.g. HR, OR) will be analysed separately.

Heterogeneity in any meta-analyses will be quantified using the I^2^ statistic to indicate variation beyond that expected from chance alone [19]. The *I*^2^ statistic has been chosen in preference to Cochran’s Q as we anticipate a small number of studies will be included in this analysis. Interpretation of heterogeneity will not simply apply a threshold judgement based on the percentage value of *I*^2^, but will also consider strength of evidence for the heterogeneity (confidence interval, chi-squared test and/or *p* value) and the size and direction of effect in the analysis [10]. The level of heterogeneity will be interpreted as follows: 0–40% (small), 30–60% (moderate), 50–90% (substantial), 75–100% (considerable) [19]. If there is substantial unaccounted heterogeneity, a meta-regression analysis will be performed.

If there are 10 or more studies included in any given meta-analysis, the potential for publication bias will be assessed by funnel plot analysis [19].

Where clinical methodological heterogeneity prevents appropriate meta-analysis of data, forest plots may be used without a summary estimate to indicate the data across studies.

### Sub-group and sensitivity analysis

Sub-group analysis will be performed to determine the impact of the following: gender, underlying aetiology, and intervening treatments (for example, renal replacement therapy V no renal replacement therapy). When performing the analysis for underlying aetiology, we will group participants according to the precipitating cause of the AKI: pre-renal, intra-renal or post-renal. Pre-renal injuries describe an obstruction of blood flow to the kidneys. This category might include surgical patients who likely experienced hypovolaemia, participants presenting with dehydration or cardiovascular participants. Intra-renal injuries describe intrinsic damage to the kidneys, and this category might include patients diagnosed with acute tubular necrosis or whose underlying cause involved an autoimmune condition such as Good pastures syndrome. Post-renal injuries describe an obstruction of urine from the kidneys and might include patients requiring urological intervention such as a nephrostomy. The information used to determine the sub-group category of the underlying aetiology will be obtained from characteristic data included in the study description or from the raw data provided by the authors.

Meta-regression may be performed to explore the size of the effect exerted by individual study variables as identified by the risk of bias and quality assessments. This may include the age of the participants, AKI duration and the duration of follow-up. Meta-regression will not be performed if there are fewer than 10 studies in a meta-analysis [19].

If there is moderate or greater quantified statistical heterogeneity in a meta-analysis and debate had previously arisen about the inclusion of all the studies, we will explore our decision-making process by removing the studies which were the source of debate. The effect of this sensitivity analysis will be reported and discussed.

In any meta-analysis performed, studies deemed at high risk of bias (judged by either the QUIPS or CHARMS method) will be excluded and the effect of this will be evaluated in a sensitivity analysis.

### Standards

Reporting will conform to Preferred Reporting Items for Systematic Reviews and Meta-Analyses (PRISMA) standards (see Additional file 3).

## Discussion

Body mass index may be an important modifiable risk factor for mortality in patients presenting with AKI. Achieving an adequate nutritional intake in sufferers with AKI is hypothesised to reduce length of stay and improve rehabilitation potential by preventing or reducing muscle wastage [20, 21]. However, the proposed benefits of body compositional preservation have never been studied. By not understanding what change in body composition is desirable for patients (depending on their presenting body composition profiles or body mass indices), renal dietitians and medical teams cannot set patient-specific nutritional goals that knowingly improve outcomes.

This proposed systematic review will help to elucidate the association between BMI and survival in patients with AKI. Differences in patient characteristics—including underlying cause of AKI; existing co-morbidities; AKI definition, severity and treatment; ethnicity; and differences in lean body mass profiles—are expected limitations of this review. The authors intend to record these characteristics and identify, where available, the factors which might influence the relationship between BMI and survival. This study also aims to highlight the heterogeneous nature of an AKI and the dangers in generalising results for all patients with an AKI.

## Additional files

Additional file 1: Data Extraction Form 1—study characteristics. (DOCX 17 kb)

Additional file 2: Modified QUIPS tool. (DOCX 24 kb)

Additional file 3: Completed PRISMA-P checklist. (DOCX 34 kb)

## Abbreviations

AKI: Acute kidney injury
ARF: Acute renal failure
BMI: Body mass index
CENTRAL: Cochrane Central Register of Controlled Trials
CHARMS: Checklist for critical Appraisal and data extraction for systematic Reviews of prediction Modelling Studies
CKD: Chronic kidney disease
GRADE: Grading of Recommendations, Assessment, Development and Evaluations
HR: Hazard ratio
KDIGO: Kidney Disease Improving Global Outcomes
OR: Odds ratio
PRISMA: Preferred Reporting Items for Systematic Reviews and Meta-Analyses
PROSPERO: International prospective register of systematic reviews
QUIPS: QUality In Prognosis Studies
RR: Relative risk
