# Supplementary material for: The impact of body mass index on mortality in patients with acute kidney injury: a systematic review protocol
Source: Syst Rev. 2018 Oct 22;7:173. doi: 10.1186/s13643-018-0825-3 (PMC6198423; doi:10.1186/s13643-018-0825-3)
Supplement: Supplementary file 1 — Data Extraction Form 1—study characteristics. (DOCX 17 kb) [file 13643_2018_825_MOESM1_ESM.docx]

Data Extraction Form 1 – Study characteristics

| **Journal citation** (Study authors, Journal name, year, volume, pages) | **study design** (observational, prognostic, prediction model) | **Setting** | **Aim of study** | **Interventions used (including treatment of AKI)** | **participant characteristics** (clinical condition, ethnicity, cause of AKI) | **inclusion/ exclusion criteria** | **AKI diagnostic criteria** | **Duration of study** | **length of follow-up** (of mortality outcome) | **BMI descriptor** (ranges used) | **Notes** |
| --- | --- | --- | --- | --- | --- | --- | --- | --- | --- | --- | --- |
|  |  |  |  |  |  |  |  |  |  |  |  |
|  |  |  |  |  |  |  |  |  |  |  |  |
|  |  |  |  |  |  |  |  |  |  |  |  |
|  |  |  |  |  |  |  |  |  |  |  |  |
|  |  |  |  |  |  |  |  |  |  |  |  |
|  |  |  |  |  |  |  |  |  |  |  |  |
|  |  |  |  |  |  |  |  |  |  |  |  |
|  |  |  |  |  |  |  |  |  |  |  |  |
|  |  |  |  |  |  |  |  |  |  |  |  |
|  |  |  |  |  |  |  |  |  |  |  |  |
|  |  |  |  |  |  |  |  |  |  |  |  |

|  | **Total number of participants** | **AKI duration** | **Number of participants with BMI < 18.5** | **Number of participants with BMI 18.5 - 24.99** | **Number of participants with BMI 25 - 29.99** | **Number of participants with Obese Class I** | **Number of participants with Obese Class II** | **Number of participants with Obese Class III** | **Timing of BMI/ body mass measurement** | **Mortality risk score** (APACHE II, CCF, HELENICC, SOFA) | **Adjustment for confounding variables used** | **Notes** |
| --- | --- | --- | --- | --- | --- | --- | --- | --- | --- | --- | --- | --- |
| AKI stage 1 |  |  |  |  |  |  |  |  |  |  |  |  |
| Mortality outcome analysis (odds ratios, hazard ratios, regression analysis) |  | NA |  |  |  |  |  |  |  |  |  |  |
| AKI stage 2 |  |  |  |  |  |  |  |  |  |  |  |  |
| Mortality outcome analysis |  | NA |  |  |  |  |  |  |  |  |  |  |
| AKI stage 3 |  |  |  |  |  |  |  |  |  |  |  |  |
| Mortality outcome analysis |  | NA |  |  |  |  |  |  |  |  |  |  |

Data Extraction Form 2 (Individual study data)
