# Supplementary material for: The impact of body mass index on mortality in patients with acute kidney injury: a systematic review protocol
Source: Syst Rev. 2018 Oct 22;7:173. doi: 10.1186/s13643-018-0825-3 (PMC6198423; doi:10.1186/s13643-018-0825-3)
Supplement: Supplementary file 2 — Modified QUIPS tool. (DOCX 24 kb) [file 13643_2018_825_MOESM2_ESM.docx]

| **Potential bias (circle one)** | | | **Items considered for assessment of potential opportunity for bias** | **Yes response** | **No repsonse** | Study 1 | Study 2 | Study 3 | Study 4 |
| --- | --- | --- | --- | --- | --- | --- | --- | --- | --- |
| **Study Population**  The study sample represents the population on key characteristics sufficient to limit potential bias to the observed relationship between BMI and mortality | | | The source population of interest is adequately described for key characteristics and the study setting supports the applicability of results. Eligibility criteria and recruitment are adequately described and the inclusion/ exclusion criteria applied uniformly to all screened for eligibility. There is adequate participation in the study by eligible individuals and sufficient information was given about those who did not participate. The baseline characteristics or participants included is adequately described for characteristics and representative of the population of interest. | AKI adult (>18 yrs) patients AND AKI criterion reported (e.g. creatinine clearance +/- urine output, staging) AND patient characteristics include ethnicity, underlying condition AND no major exclusions AND treatment method for AKI (if applicable) described e.g. RRT, mechanical ventilation | Non-adults included, patient characteristics not adequately described, underlying condition unknown/ not detailed, major exclusions involved and treatment method not detailed |  |  |  |  |
|  |  |  |  |  |  |  |  |  |  |
|  |  |  |  |  |  |  |  |  |  |
|  |  |  |  |  |  |  |  |  |  |
|  |  |  |  |  |  |  |  |  |  |
| Yes | Partly | No |  |  |  |  |  |  |  |
| **Study Attrition**  Loss to follow-up (from sample to study population) is not associated with key characteristics (i.e. the study data represent the sample), sufficient to limit potential bias | | | Attempts to collect information on participants who dropped out of the study are described. Reasons for loss to follow-up are provided. There are no important differences between key characteristics (e.g. ethnicity, underlying condition, age, treatment method) and outcomes in participants who completed the study and those who did not. | Reasons lost to follow-up reported with numbers AND comparison of lost verses not lost to follow-up with no important differences, or if important differences found addressed in the analysis | Attrition/ denominators not reported/ accounted for |  |  |  |  |
|  |  |  |  |  |  |  |  |  |  |
|  |  |  |  |  |  |  |  |  |  |
|  |  |  |  |  |  |  |  |  |  |
|  |  |  |  |  |  |  |  |  |  |
| Yes | Partly | No |  |  |  |  |  |  |  |
| **Prognostic factor measurement**  BMI/ body mass is adequately measured in study participants to sufficiently limit bias | | | Body mass measured at time of AKI presentation. Clear definition of BMI given and BMI category ranges provided with number of participants in each BMI category reported OR sufficient data provided to determine BMI categories. Adequate proportion of the study sample has complete data. | Data collection is prospective and recorded on presentation of AKI AND BMI criteria defined | Definition of BMI not clear or sufficiently detailed (e.g. BMI > 30 is categoried as obese with no other obese categories included or clear data on the range of BMIs > 30) |  |  |  |  |
|  |  |  |  |  |  |  |  |  |  |
|  |  |  |  |  |  |  |  |  |  |
|  |  |  |  |  |  |  |  |  |  |
| Yes | Partly | No |  |  |  |  |  |  |  |
| **Outcome measurement** | | | Clear definition of mortality measurement provided, including duration of follow-up. Mortality risk measured prior to outcome occurring. | Mortality incidence recorded AND time-frame for mortality reported AND data collection for mortality risk is prospective | mortality incidence and time-frame for follow-up not reported |  |  |  |  |
| Mortality incidence is adequately measured in study participants to sufficiently limit potential bias | | |  |  |  |  |  |  |  |
|  |  |  |  |  |  |  |  |  |  |
|  |  |  |  |  |  |  |  |  |  |
|  |  |  |  |  |  |  |  |  |  |
| Yes | Partly | No |  |  |  |  |  |  |  |
| **Confounding measurement and account**  Important potential confounders are appropriately accounted for, limiting potential bias with respect to body mass | | | Important potential confounders are accounted for in the study design (e.g. patients separated into different BMI categories if ethnic origin differs i.e. afro-carribean, south asian) and analysis. Measurement of all important confounders is adequately valid and reliable (e.g. accounting for hydration status when measuring weight -- under-hydrated or fluid overloaded patients). The method and setting of confounding measurement are the same for all participants (e.g. same scales). Appropriate imputation method is used for missing confounder data. Appropriate adjustment used and clearly outlined. Interventions do not confound body mass results or mortality outcome. | BMI categories adequately defined AND based on accurate, reliable weights. Adjusters, if used, are appropriate and clearly outlined. Intervention method does not impact on mortality outcome | BMI or body mass measurements are not clearly or adequately defined. Validity of body mass measurements not reported on |  |  |  |  |
|  |  |  |  |  |  |  |  |  |  |
|  |  |  |  |  |  |  |  |  |  |
|  |  |  |  |  |  |  |  |  |  |
|  |  |  |  |  |  |  |  |  |  |
| Yes | Partly | No |  |  |  |  |  |  |  |
| **Analysis and reporting** | | | There is sufficient presentation of data to assess the adequacy of the analysis. The selected statistical method of analysis is adequate for the design of the study (e.g. mortality incidence risk). There is no selective reporting of results. | Statistical model used appropriate for the study design and type of data AND strategy and results clearly reported AND completeness of reporting of results | Unclear reporting of strategy or results AND inappropriate statistical model AND selective reporting of results |  |  |  |  |
| The statistical analysis is appropriate for the design of the study, limiting potential for presentation of invalid results | | |  |  |  |  |  |  |  |
|  |  |  |  |  |  |  |  |  |  |
|  |  |  |  |  |  |  |  |  |  |
|  |  |  |  |  |  |  |  |  |  |
| Yes | Partly | No |  |  |  |  |  |  |  |
